# Supplementary material for: Replica molding-based nanopatterning of tribocharge on elastomer with application to electrohydrodynamic nanolithography
Source: Nat Commun. 2018 Mar 2;9:974. doi: 10.1038/s41467-018-03319-4 (PMC5834498; doi:10.1038/s41467-018-03319-4)
Supplement: Supplementary file 1 — Supplementary Information [file 41467_2018_3319_MOESM1_ESM.pdf]

Supplementary Information

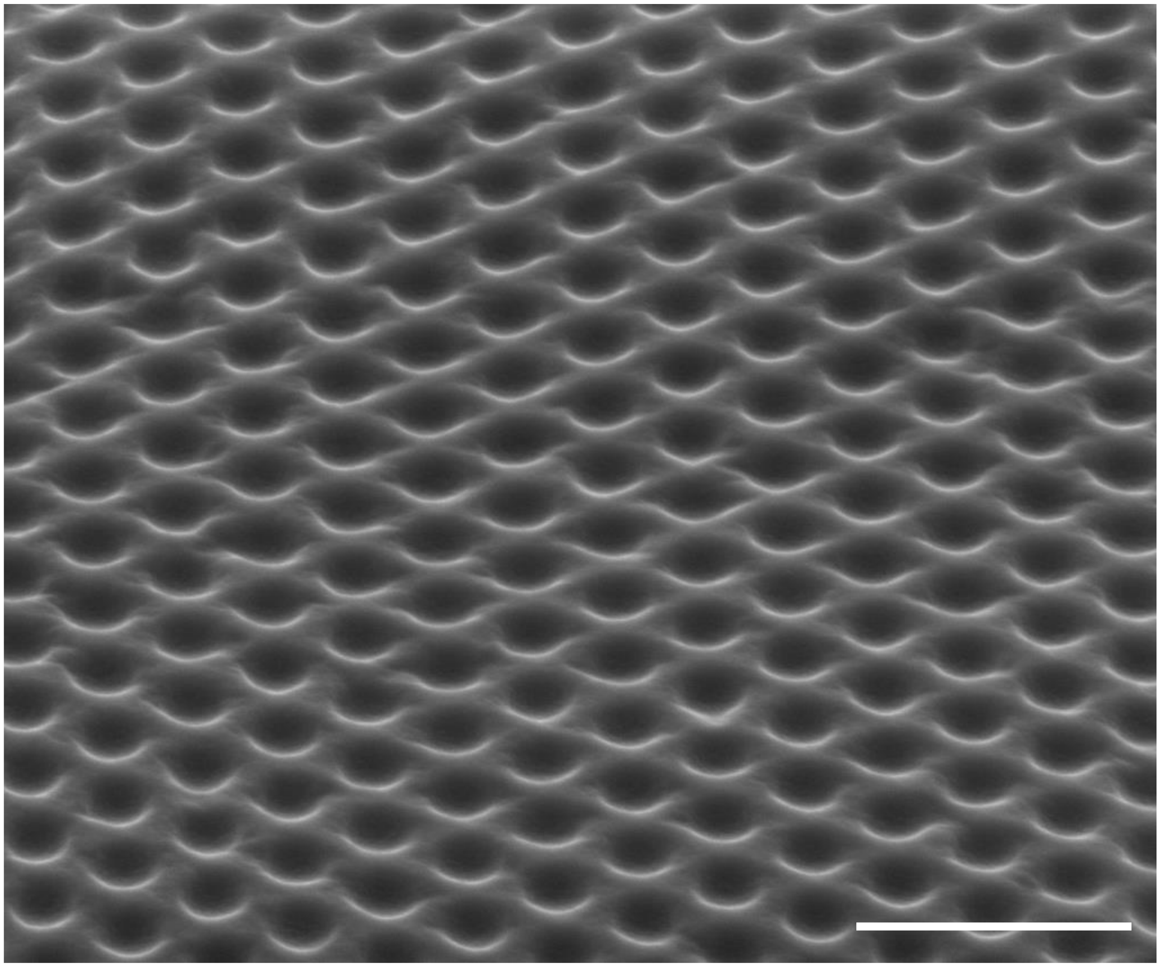

Supplementary Figure 1 Morphology of PDMS nanocups This scanning electron micrograph of the PDMS nanocups, taken with the sample stage tilted by ~55°, clearly shows the regularly arrayed apertures of the nanocups and the flat interstitial area between them. Scale bar: 2 μm.

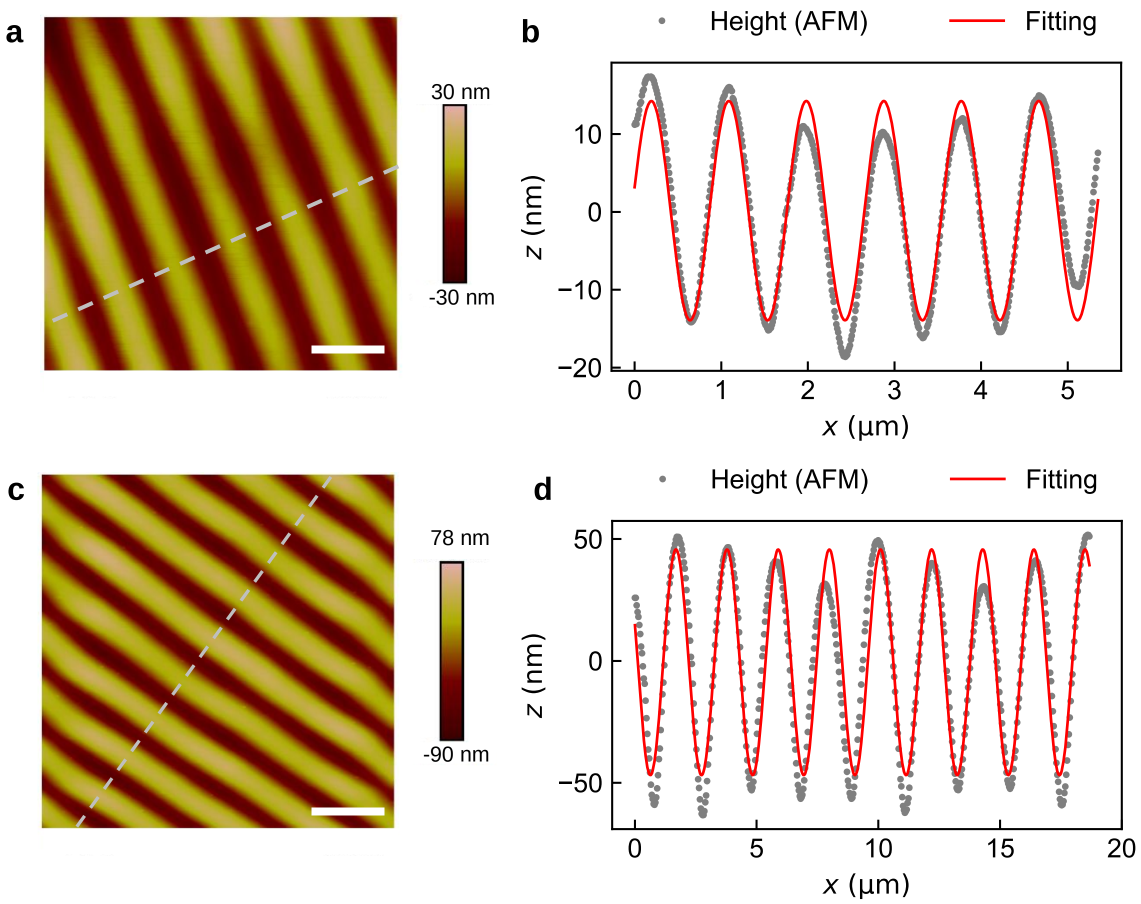

Supplementary Figure 2 Morphology of UV-induced sinusoidal textures These atomic force micrographs show the morphologies of the UV 2-beam interference-induced sinusoidal textures made on NOA73. (**a**) and (**b**) show the top and profile views of a texture with 900 nm pitch (10° tilt angle in the Lloyd mirror setup) and 29±2.7 (s.d.) nm in depth. The dose and exposure time were 1.6 J cm^{-2} and 60 mins, respectively. (**c**) and (**d**) are from another texture with 2.1 μm pitch (2° tilt angle) and 99±11 (s.d.) nm in depth. The dose and exposure time were 2.2 J cm^{-2} and 80 mins, respectively. The laser intensity was ~0.45 mW cm^{-2}. In **b** and **d**, the red solid curves represent sinusoidal fitting results, which confirm the sine-squared-nature of the interference intensity pattern in Lloyd setup. Scale bars: 1 μm and 3 μm for **a** and **c**, respectively.

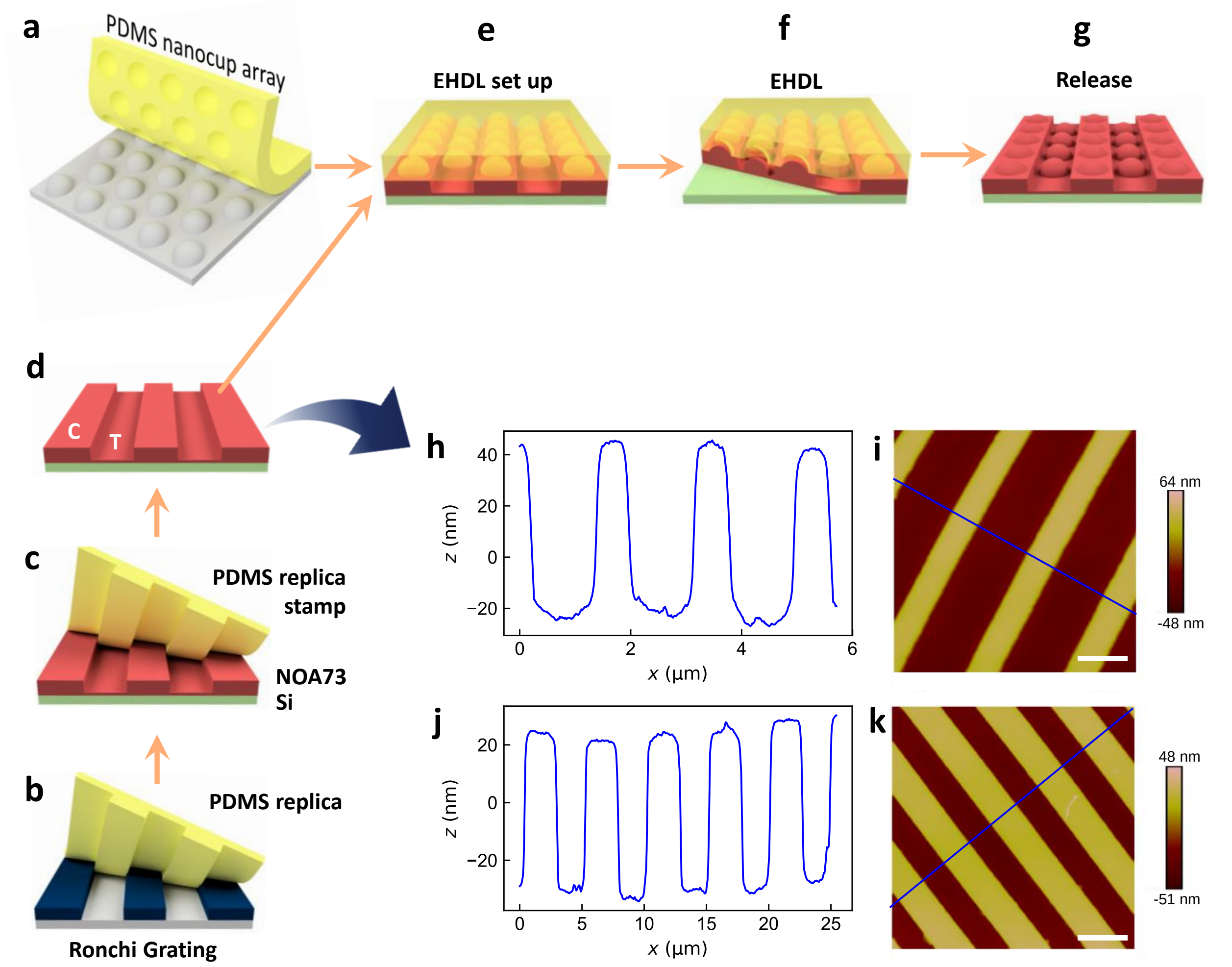

Supplementary Figure 3 Tribocharge-enabled EHDL on NOA73 surfaces corrugated through replica molding (**a**) Liquid-phase PDMS is poured onto the PC mold textured with a 2D triangular nanocone array. After thermal curing, the PDMS replica, textured with a nanocup array, is peeled off. Its surface becomes selectively tribocharged during the demolding process. (**b**) A PDMS mold is replicated from Ronchi gratings. (**c**) The PDMS replica is placed in contact with the spin-coated NOA73 film. (**d**) The linearly corrugated NOA73 film is obtained after the partial curing of the NOA73 with UV light and removal of the PDMS replica. (**e**) The tribocharged PDMS nanocup array is placed on the textured NOA73 film. (**f**) NOA73 in the trough region is attracted upward by the spatially modulated electric fields originated from the tribocharges and undergoes EHDL. (**g**) The final UV-induced solidification of NOA73 and removal of the PDMS nanocup array complete the tribocharge-enabled EHDL of NOA73. (**h**) The line profile along the solid line in the AFM image (**i**) of NOA73 surface with a 1.7 µm-pitch linear corrugation (Scale bar: 1 µm). (**j**) The line profile along the solid line in the AFM image (**k**) of NOA73 surface with a 5 µm-pitch linear corrugation (Scale bar: 4 µm).

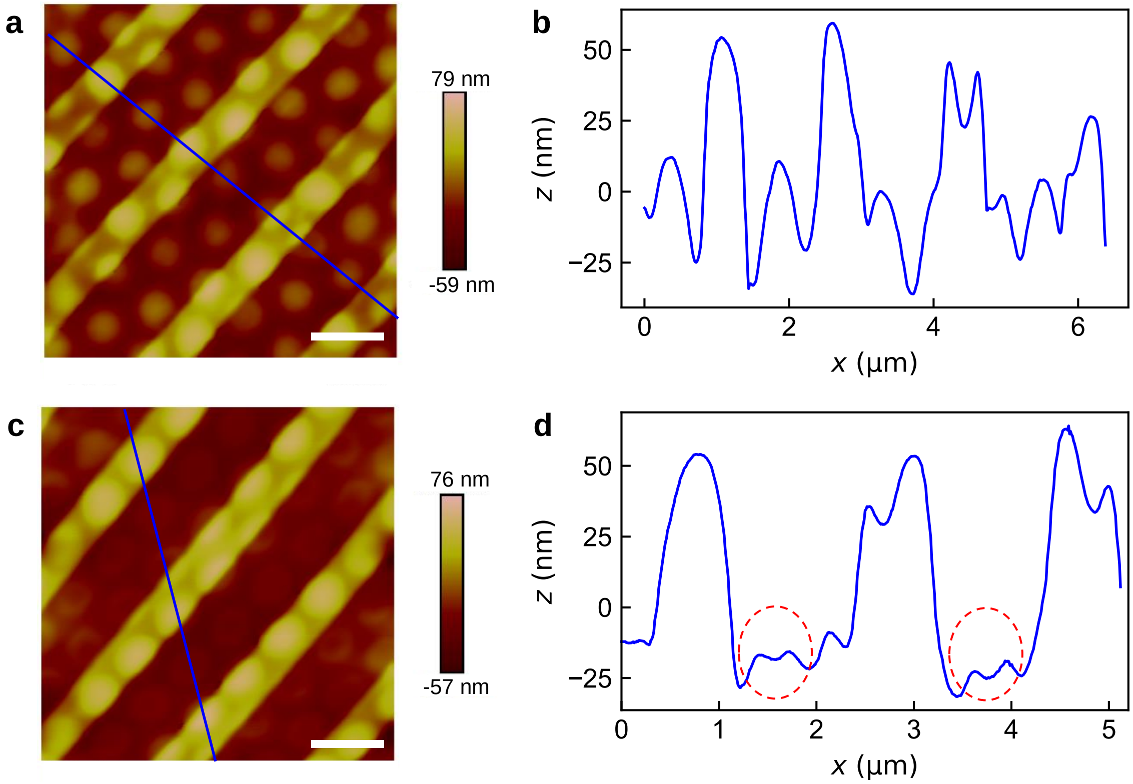

Supplementary Figure 4 Tribocharge-enabled EHDL on NOA73 surface with a 1.7 μm-pitch linear corrugation (**a**-**d**) show the results of performing tribocharge-enabled EHDL on an NOA73 surface textured with replica molding and partial UV curing, rather than the UV laser two-beam interference adopted in the main text. **a** and **b** are made with 120 s exposure under 15 mW cm^{-2} intensity, or a dose of 1.8 J cm^{-2}. **c** and **d** are made with 140 s exposure under 15 mW cm^{-2} intensity, or a dose of 2.1 J cm^{-2}. In the trough of **d**, which is more viscous due to the higher dose, the formation of nanovolcano is observed (dotted circles). (Scale bars: 1 μm).

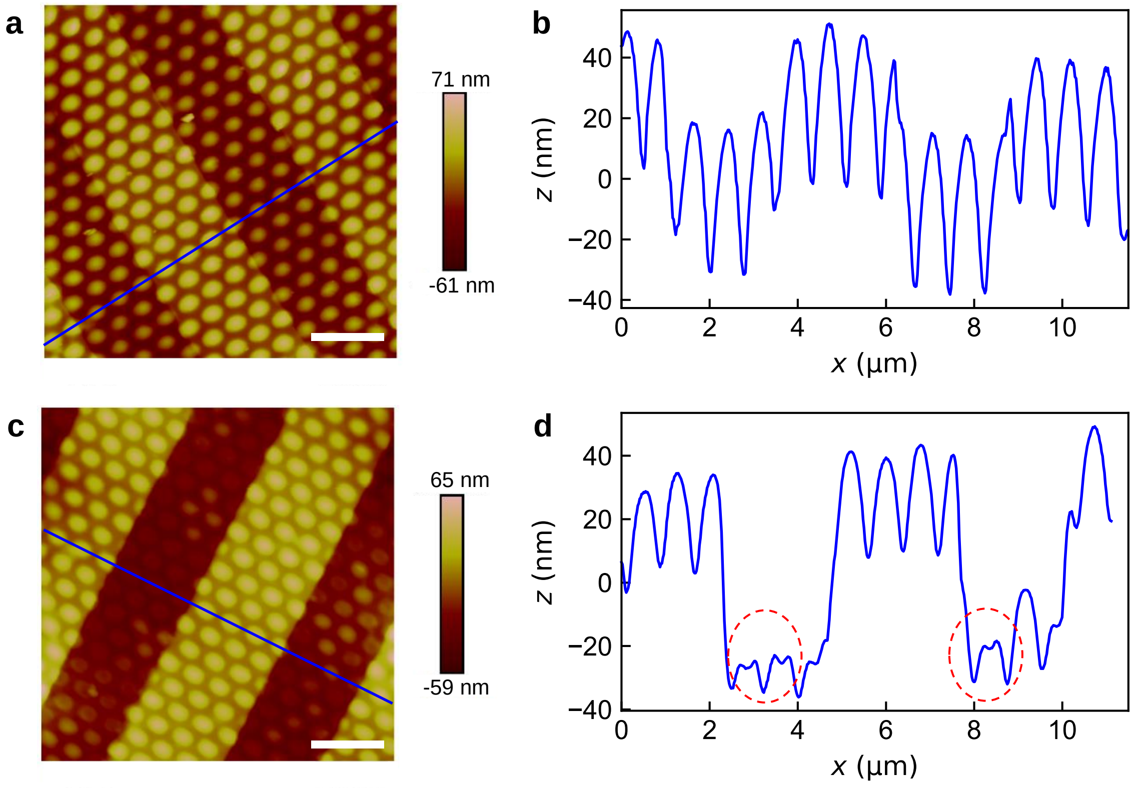

Supplementary Figure 5 Tribocharge-enabled EHDL on NOA73 surface with 5 μm-pitch linear corrugation (**a**-**d**) show the results of performing tribocharge-enabled EHDL on an NOA73 surface textured replica molding and partial UV curing, rather than the UV laser two-beam interference adopted in the main text. **a** and **b** are made with 90 s exposure under 15 mW cm^{-2} intensity, or a dose of 1.35 J cm^{-2}. **c** and **d** are made with 120 s exposure under 15 mW cm^{-2} intensity, or a dose of 1.8 J cm^{-2}. In the trough of **d**, which is more viscous due to the higher dose, the formation of nanovolcano is observed (dotted circles). (Scale bars: 2 μm).

Supplementary Note 1

Tribocharge-enabled EHDL on NOA73 surface with linear corrugation

To further validate the working principle of the tribocharge-enabled EHDL and its robustness, we repeated the process in a modified setup and checked if the nanovolcanos could still be formed. Specifically, we tried to induce the nanovolcano formation on an NOA73 surface with linear corrugations, instead of the sinusoidal ones formed with the two-beam interference.

The preparation steps are shown in Supplementary Fig. 3. First, a PDMS mold was replicated from Ronchi gratings (600 LPMM, MaxLevy; 200 LPMM, Edmund Optics) (Supplementary Fig. 3b). Then the PDMS mold was placed in contact with the spin-coated NOA73 film (Supplementary Fig. 3c) and peeled off after the NOA73 film was partially cured under the broadband UV light (Bluewave 200, Dymax) at 15 mW cm^{-2} for a preset period of time (Supplementary Fig. 3d) and examined by AFM (Supplementary Figs. 3h and 3i). Owing to the high oxygen permeability of PDMS and the intrinsic oxygen inhibitory nature of NOA73, the top layer of the NOA73 surface remained fluidic and patternable. In addition, the NOA73 surface was uniformly cured in this scenario since the amplitude of the corrugation (around 60 nm) is much smaller than the thickness of the PDMS mold (2~3 mm). The tribocharged PDMS mold with nanocups was later placed in contact with the partially cured NOA73 surface to induce the tribocharge-enabled EHDL (Supplementary Figs. 3e and 3f). Upon its complete curing and detachment from the PDMS mold, the NOA73 structure was AFM scanned (Supplementary Figs. 3g).

Using samples prepared through such a disparate procedure, we tried to test whether

(1) The tribocharge-enabled EHDL works,
(2) The UV dose-controlled switching between nanocone and nanovolcano works.

Supplementary Fig. 4 shows the result obtained from the NOA73 surface pre-textured at 1.7 µm pitch. The upper row (a and b) corresponds to lower dose exposure (1.8 J cm^{-2}) and the lower row (c and d) corresponds to higher dose exposure (2.1 J cm^{-2}). As emphasized by the dotted circles in Supplementary Fig. 4d, the formation of center dimples and, hence, nanovolcanos occurred only for higher UV dose, higher viscosity case.

The trend was repeated in Supplementary Fig. 5 which was obtained from the NOA73 surface pre-textured at a wider, 5.0 µm pitch. Still, the upper row (a and b) corresponds to lower dose exposure (1.35 J cm^{-2}) and the lower row (c and d) corresponds to higher dose exposure (1.8 J cm^{-2}). The dotted circles in Supplementary Fig. 5d indicate that the nanovolcano formation occurred only in the higher viscosity sample prepared under higher UV dose.

Therefore, both (1) and (2) got checked out successfully. Higher UV dose did lead to the formation of nanovolcanos in the trough despite a variety of changes made to the setup. These results re-affirm the validity and robustness of the nanovolcano formation mechanism.

It is worth noting that in the two-beam interference-based EHDL, nanovolcanos were formed even in the crest region as shown in Fig. 4i (red trace). Although their nanocraters are lower than those in the trough region, such a nanovolcano formation contrasts the results in Supplementary Figs. 4 and 5 in which the crest portion is occupied only by nanocones. We ascribe the discrepancy to a possible incomplete destructive interference. Even though the optical table was floated, the long exposure time (~60 minutes) may have imparted residual UV dose on the crest region, rendering the area more viscous and less deformable. Since the crest area is in contact with the PDMS nanocups, the main mechanism for its shaping is capillary action. The reduction in the fluidicity, in combination with the Coulombic attraction from the rim charges mentioned in the "Underfilled crest nanocone as ring charge evidence" subsection of the main text, could impede the complete filling of the nanocup and the formation of perfect nanocones, leaving them with small dips as shown in Fig. 4i. The higher UV dose (~3.6 $J \cdot cm^{-2}$ as opposed to 1.3~2.1 $J \cdot cm^{-2}$ in Supplementary Figs. 4 and 5) could have aggravated the process. Eventually, they could lead to an incomplete nanocone formation, i.e., the appearance of the shallow dip at the center.
