# Supplementary material for: Replica molding-based nanopatterning of tribocharge on elastomer with application to electrohydrodynamic nanolithography
Source: Nat Commun. 2018 Mar 2;9:974. doi: 10.1038/s41467-018-03319-4 (PMC5834498; doi:10.1038/s41467-018-03319-4)
Supplement: Supplementary file 3 — Description of Additional Supplementary Files [file 41467_2018_3319_MOESM3_ESM.pdf]

Description of Additional Supplementary File

File Name: Supplementary Movie 1
Description: **Finite element analysis of dynamic demolding process**. This movie shows the formation and spreading of the frictional stress σ_f (Pa) along the progress of the demolding action. The overall setup is identical to that of Figs. 6a-c. The frictional stress initially develops near L~0.5·L_tot but quickly spreads to the rim area to form a strip with its maximum near L~0.15·L_tot. Interestingly, the rim itself, which forms the upper boundary of the strip, suffers frictional stress far lower than that at the center of the strip. These trends in the temporal change of the frictional level agree well with the cumulative impact shown in the form of the frictional fracture energy G_f (J m^{-2}) in Fig. 6d.
